# Fractional Flow Reserve Evaluated as Metric of Coronary Stenosis — A Mathematical Model Study

**DOI:** 10.3389/fcvm.2019.00189

**Published:** 2020-01-14

**Authors:** Theo J. C. Faes, Romain Meer, Guy R. Heyndrickx, Peter L. M. Kerkhof

**Affiliations:** ^1^Department of Radiology and Nuclear Medicine, Amsterdam University Medical Centers, Amsterdam, Netherlands; ^2^Cardiovascular Center, Aalst, Belgium

**Keywords:** coronary circulation, coronary stenosis, degree of stenosis, fractional flow reserve, mathematical model, clinical metrics, *in silico* study

## Abstract

**Introduction:** Coronary arterial stenosis may impair myocardial perfusion with myocardial ischemia and associated morbidity and mortality as result. The myocardial fractional flow reserve (*FFR*) is clinically used as a stenosis-specific index.

**Aim:** This study aims to identify the relation between the *FFR* and the degree of coronary arterial stenosis using a simple mathematical model of the coronary circulation.

**Methods:** A mathematical model of the coronary circulation, including an arterial stenosis of variable degree, was developed. The relation between the *FFR* and the degree of stenosis (defined as the fractional cross sectional area narrowing) was investigated, including the influence of the aortic and venous pressures and the capillary resistance. An additional study concerning 22 patients with coronary artery disease permits comparison of clinical data and *in silico* findings.

**Results:** The *FFR* shows an S-shaped relationship with the stenosis index. We found a marked influence of venous and aortic pressure and capillary resistance. The *FFR* is accompanied by a clinically relevant co-metric (*FFR*_*C*_), defined by the Pythagorean sum of the two pressures in the definition formula for *FFR*. In the patient group the *FFR*_*C*_ is strongly related to the post-stenotic pressure (*R* = 0.91). The *FFR*_*C*_ requires establishment of a validated cut-off point using future trials.

**Conclusion:** The S-shaped dependence of *FFR* on the severity of the stenosis makes the *FFR* a measure of the ordinal scale. The marked influences of the aortic and venous pressures and the capillary resistance on the *FFR* will be interpreted as significant variations in intra- and inter-individual clinical findings. These fluctuations are partly connected to the neglect of considering the *FFR*_*C*_. At otherwise identical conditions the *FFR* as measured at baseline differs from the value obtained during hyperemic conditions. This expected observation requires further investigation, as the current hyperemia based evaluation fails to take advantage of available baseline data.

## Introduction

A coronary artery stenosis may seriously affect myocardial perfusion with myocardial ischemia or even cardiac death as possible sequelae (1). Consequently, a function limiting coronary arterial stenosis is associated with a significant increase in morbidity and mortality, although the underlying mechanisms may partly differ for men and women (2–5). Traditionally, a stenosis has been evaluated by angiography leading to a preoccupation with coronary luminology (6). Inadequacy of this method led to a number of alternative approaches, both invasive such as pressure determinations, and non-invasive techniques including Doppler echocardiography. Often the fractional flow reserve (*FFR*) is clinically used as a stenosis-related index of maximum attainable local myocardial perfusion. By using pressure wires, the *FFR* is assessed by measuring invasively the coronary pressures proximal and distal of the stenosis. The *FFR* is defined as the ratio of mean coronary pressures measured directly distal and proximal of the stenosis, i.e., *FFR* = P_Distal_/P_Proximal_ [a dimensionless number in the numerical range from 0 (complete occlusion) through 1 (no occlusion)]; an *FFR*-value below 0.80 is considered to reflect a clinically significant stenosis. The *FFR* is thought to be a stenosis-specific index that reflects the effect of the coronary stenosis on the myocardial perfusion. Moreover, the *FFR* is reported to be independent of hemodynamic characteristics of the coronary circulation, such as blood pressure, heart rate, and cardiac contractility (7). In clinical practice, however, the use of the *FFR* is somewhat limited by the high costs, the extra time involved, and the need to administer adenosine to induce hyperemia with an associated risk and burden for the patient. Furthermore, the ratio *FFR* does not address its intrinsic companion, being the Pythagorean sum of P_Distal_ and P_Proximal_ (8) (see section Methods for details).

By using a simple mathematical model of the coronary circulation, this study aims to identify the relationship of the *FFR* on the degree of stenosis, while evaluating hemodynamic characteristics of the arterial coronary circulation. Recently, Duanmu et al. presented a lumped-parameter model of the coronary circulation (9). In their model, the coronary circulation is described by defining a number of compartments, and by characterizing the hemodynamics of each compartment with the use of a three-element Windkessel model (10). Each Windkessel consists of a dissipative Poiseuille resistance (*R*), a blood storing compliance (*C*), and a blood mass inertance (*L*). The values of these three elements (*R, C, L*) were calculated from the length and diameter of the coronary vessel using CT-images (9). To study the effect of a stenosis on the coronary hemodynamics and the *FFR*, we extended their model by including an extra dissipative resistance to more precisely mimic the stenosis. The focus of our present study is more limited than explored in Duanmu's model. Our plain model is used to gain insight in the fundamental characteristics of the *FFR* metric. This discernment will guide our future simulation studies employing the Duanmu model as a convenient starting point for both the left and right coronary artery.

Our present study aims are:

- To discuss the *FFR*'s definition with its assumptions and their theoretical consequences;
- To identify the relation of the *FFR* and the underlying degree of coronary arterial stenosis;
- To discuss the consequences of defining the *FFR* as a ratio of two pressures;
- To hint for an alternative for the *FFR*.

For verification of the outcomes, the theoretical issues will be related to available clinical data.

## Methods

The *FFR* is determined by measuring the mean aortic pressure, $P_{A}^{\left(H\right)}$, and the mean pressure distal to the stenosis, $P_{D}^{\left(H\right)}$, during hyperemia, and by the subsequent calculation of the ratio *FFR* = $P_{D}^{\left(H\right)}$/$P_{A}^{\left(H\right)}$. In the following we will discuss: firstly, the rationale of *FFR* as ratio of distal-to-stenosis pressure to aortic pressure and, secondly, the mathematical relationship between this *FFR* and the stenosis's geometry (i.e., the ratio of the stenotic cross sectional area to the non-stenotic area) as determinant of the flow limitation.

### The Rationale of the FFR as Pressure Ratio

In their landmark study Pijls and De Bruyne start from the definition of *FFR* as the maximum myocardial blood flow in the presence of a stenosis, *F*_*Smax*_, divided by the theoretical maximum myocardial blood flow in the absence of the stenosis, *F*_*Nmax*_. Subsequently, they show that the *FFR* is approximately equal to the ratio of the mean pressure measured distal to the stenosis, $P_{D}^{\left(H\right)}$, and the mean aortic pressure $P_{A}^{\left(H\right)}$ both measured during hyperemia (as indicated by the letter *H* in the superscript), i.e.,

(1) $$\begin{array}{l} FFR=\frac{F_{S_{MAX}}}{F_{N_{MAX}}}\approx \frac{P_{D}^{\left(H\right)}}{P_{A}^{\left(H\right)}} \end{array}$$

The maximum flows are achieved by the administration of a hyperemic agent, e.g., adenosine.

**Figure 1:**
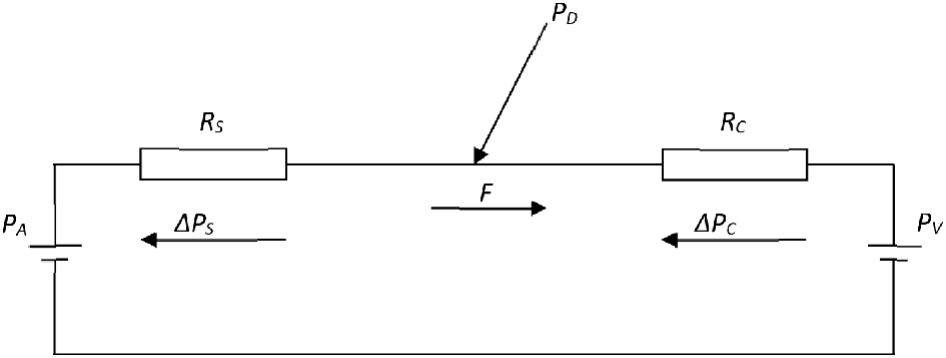
Electrical analog of the coronary circulation. The model consists of an aortic pressure *P*_*A*_ and a venous pressure *P*_*V*_ with two resistances, serially connected between these pressure sources, to represent the stenotic *R*_*S*_ and capillary *R*_*C*_ fluid resistances. *F* is the flow through the resistances, while Δ*P*_*S*_ and Δ*P*_*C*_ are the pressure drops over the resistances *R*_*S*_ and *R*_*C*_, respectively. *P*_*D*_ is the pressure distal to the stenosis and proximal to the capillaries.

To accomplish this result, Pijls and De Bruyne use a two-compartment model to characterize the blood flow in the vascular bed of the coronary circulation: one compartment represents the coronary arteries, with or without stenosis, and the second compartment represents the remaining distal capillary network and venous vessels of the coronary circulation (Figure 1). This model defines the following quantities: Let *P*_*A*_ and *P*_*V*_ be the mean aortic and the venous pressure (mmHg), respectively, and let *P*_*D*_ be the mean pressure (mmHg) distal of the epicardial artery stenosis. Moreover, let *R*_*A*_ and *R*_*C*_ be the hemodynamic resistances (mmHg.s/ml) of the arterial part (either non-stenotic or stenotic) and the capillary and venous part, respectively. Finally, after the administration of a hyperemic agent the capillary resistance reduces to *R*_*C*__min_ and the flow increases to *F*_max_, while the distal pressure *P*_*D*_ decreases to $P_{D}^{\left(H\right)}$. Within this model, simple hemodynamic reasoning yields the following results:

1. By application of Poiseuille's law (i.e., flow equals pressure drop divided by fluid resistance; see Equation 11) to the capillary compartment, the baseline and maximum flows equal the perfusion pressures *P*_*D*_ – *P*_*V*_ and $P_{D}^{\left(H\right)}$ – *P*_*V*_ divided by the capillary hemodynamic resistance *R*_*C*_ and *R*_*C*__min_, respectively. That is,
  (2) $$\begin{array}{l} F=\frac{P_{D}-P_{V}}{R_{C}}\;\&\;F_{MAX}=\frac{P_{{\;}_{D}}^{\left(H\right)}-P_{V}}{R_{C_{MIN}}} \end{array}$$
2. For the non-stenotic case, the pressure drop over the arterial part is negligible compared to the capillary pressure drop, so, *P*_*D*_ ≈ *P*_*A*_ >> *P*_*V*_ and $P_{D}^{\left(H\right)}$ ≈ $P_{A}^{\left(H\right)}$ >> *P*_*V*_ (where >> indicates: much larger). Hence, by substitution of $P_{D}^{\left(H\right)}$ ≈ *P*_*A*_ in Equation (2), the non-stenotic flow *F*_*N*_ becomes,
  (3) $$\begin{array}{l} \;F_{N}=\frac{P_{D}-P_{V}}{R_{C}}\approx \frac{P_{A}-P_{V}}{R_{C}}\;\&\\ F_{N_{MAX}}=\frac{P_{{\;}_{D}}^{\left(H\right)}-P_{V}}{R_{C_{MIN}}}\approx \frac{P_{A}^{\left(H\right)}-P_{V}}{R_{C_{MIN}}}\approx \frac{P_{A}-P_{V}}{R_{C_{MIN}}} \end{array}$$
  assuming an unchanged aortic pressure during hyperemia.
3. For the stenotic case, the pressure drop over the arterial part is non-negligible compared to the capillary pressure drop, so, *P*_*A*_ > *P*_*D*_ > *P*_*V*_ and $P_{A}^{\left(H\right)}$ > $P_{D}^{\left(H\right)}$ > *P*_*V*_. Hence, with Equation (2), the stenotic flow *F*_*S*_ becomes,
  (4) $$\begin{array}{l} F_{S}=\frac{P_{D}-P_{V}}{R_{C}}\;\&\;F_{S_{MAX}}=\frac{P_{{\;}_{D}}^{\left(H\right)}-P_{V}}{R_{C_{MIN}}} \end{array}$$
4. By substitution of these flows (Equations 3 and 4) in the definition of *FFR* (Equation 1) yields,
  (5) $$\begin{array}{l} FFR\;\overset{\left(1\right)}{=}\;\frac{F_{S_{MAX}}}{F_{N_{MAX}}}\;\overset{\left(2\right)}{=}\;\frac{\frac{P_{D}^{\left(H\right)}-P_{V}}{R_{C_{MIN}}}}{\frac{P_{A}^{\left(H\right)}-P_{V}}{R_{C_{MIN}}}}\;\overset{\left(3\right)}{=}\;\frac{P_{D}^{\left(H\right)}-P_{V}}{P_{A}^{\left(H\right)}-P_{V}}\;\overset{\left(4\right)}{\approx }\;\frac{P_{D}^{\left(H\right)}}{P_{A}^{\left(H\right)}} \end{array}$$

with the following rationale applied at the numbered signs of equality: (1) definition of *FFR* (Equation 1); (2) substitution of Equations (3, 4); (3) cancelation of the common term *R*_*C*__min_; (4) neglecting *P*_*V*_, as considered small compared to $P_{D}^{\left(H\right)}$ and *P*_*A*_.

Note that, occasionally, the *FFR* is measured as the ratio of the distal-to-stenosis pressure and the aortic pressure in *baseline* (B) instead of the *hyperemic* state. Let *FFR*^(*H*)^ and *FFR*^(*B*)^ refer to hyperemic and baseline state, respectively. Then, Equation (5) implies that *FFR*^(*H*)^ ≤ *FFR*^(*B*)^ since

(6) $$\begin{array}{l} FFR^{\left(H\right)}\;=\;\frac{P_{D}^{\left(H\right)}}{P_{A}^{\left(H\right)}}\;\approx \;\frac{P_{{\;}_{D}}^{\left(H\right)}}{P_{A}}\;\leq \;\frac{P_{{\;}_{D}}^{\left(B\right)}}{P_{A}}\;=\;FFR^{\left(B\right)} \end{array}$$

as hyperemic $P_{D}^{\left(H\right)}$ is lower than the baseline $P_{D}^{\left(B\right)}$ and as hyperemic and baseline aortic pressure are approximately equal, $P_{A}^{\left(H\right)}$ ≈ *P*_*A*_. Thus, the *FFR* during hyperemia is smaller than *FFR* during baseline.

To summarize: under the assumptions of the model with Poiseuillean resistances, the *FFR* as ratio of the maximum stenotic blood flow and the theoretical maximum non-stenotic blood flow is approximated by the ratio of the mean hyperemic post-stenotic pressure $P_{D}^{\left(H\right)}$ and the mean aortic pressure $P_{A}^{\left(H\right)}$.

### Consequences of Defining the *FFR* as Ratio of Pressures

From a mathematical-physiologic viewpoint, one might interpret the *FFR* as a summary of two pressures, *P*_*D*_ and *P*_*A*_, in only one number, being the ratio *P*_*D*_/*P*_*A*_. Subsequently, one might wonder whether relevant information is lost by summarizing two pressures in a single number which concerns a dimensionless ratio.

**Figure 2:**
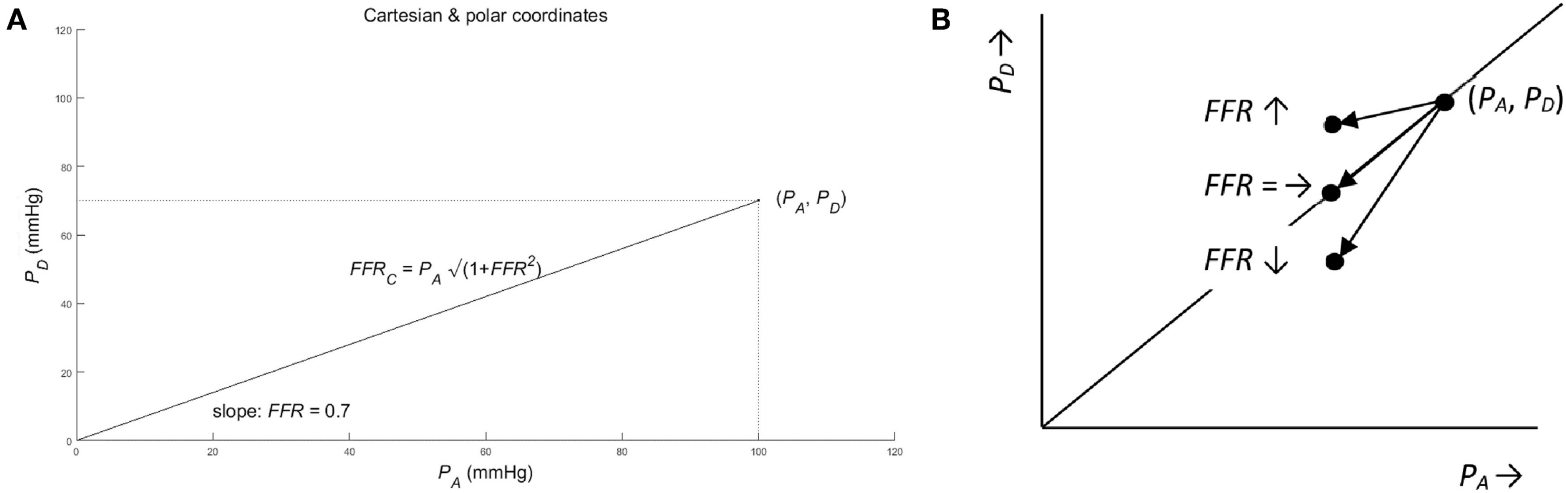
**(A)** Cartesian and polar coordinates. The pressure *P*_*A*_ and *P*_*D*_ represented in Cartesian coordinates (*P*_*A*_, *P*_*D*_) (see dotted lines) and in polar coordinates ($P_{A}\sqrt{\left(1+FFR^{2}\right)},FFR)$. Note that the pair of pressure (*P*_*A*_, *P*_*D*_) define exactly one point in the graph, while the single *FFR* defines a complete line in the graph. So, mathematically speaking, the single *FFR* without the companion *FFR*_*C*_ leaves ambiguity. As a result, all points on a line through the origin share the same value of *FFR*. (For details see Methods section). **(B)** Ambiguous change of *FFR* from point (*P*_*A*_, *P*_*D*_) for three different cases of a pressure decrease: The *FFR* is unchanged (see notation *FFR* =), if the change in *P*_*D*_ is equal to the change of *P*_*A*_ times *FFR*. Similarly, the *FFR* decreases (see FFR ↓) and increases (see FFR ↑), if the change in *P*_*D*_ is smaller and larger than the change of *P*_*A*_ times *FFR*, respectively (see section Methods for details).

A convenient way to analyze the consequences of using the ratio, is to employ the analogy with the Cartesian and polar coordinate systems (see Figure 2). To be specific, consider the pressures (*P*_*A*_, *P*_*D*_) as a point in a graph with *P*_*A*_ and *P*_*D*_ on the abscissa and ordinate (horizontal and vertical axis), respectively. Hence, (*P*_*A*_, *P*_*D*_) act as the Cartesian coordinates. Alternatively, the same point can be defined by the polar coordinates: (i) the length of the line from the origin to the point (*P*_*A*_, *P*_*D*_); (ii) the angle between this line and the positive abscissa (or equivalently by the slope of this line). By using the Pythagorean theorem, the line's length, say *FFR*_*C*_, equals √(*P*_*A*_^2^ + *P*_*D*_^2^) and the tangent or slope of the angle *P*_*D*_/*P*_*A*_ equals *FFR* (see Figure 2). Hence, *FFR*_*C*_ = √(*P*_*A*_^2^ + *P*_*D*_^2^) and *FFR* act as the polar coordinates. So, in the graph the point is characterized complete by either the Cartesian coordinates (*P*_*A*_, *P*_*D*_) or the polar coordinates *FFR*_*C*_ = √(*P*_*A*_^2^ + *P*_*D*_^2^) and *FFR* = *P*_*D*_^/^*P*_*A*_.

For later use, note the following mathematical relations:

(7) FFR=PDPA ⇒ PD=PA FFR and PA=1FFRPD         FFRC=PA2+PD2=PA2+PA2FFR2=                    PA1+FFR2≈PA(1+12FFR)

where the approximation of the square root results from Newton's Binomial Series, and becomes more accurate for smaller values of *FFR*. Note: if two out of the four variables *P*_*D*_, *P*_*A*_, *FFR, FFR*_*C*_ are known, then the remaining variables can be calculated.

Using these two coordinate systems, the original question—whether information is lost by using the *FFR*—can be answered from a mathematical point of view. By using the *FFR*, as a summary measure of *P*_*A*_ and *P*_*D*_, information is clearly lost, because only one of the two polar coordinates is used while the other polar coordinate is neglected. Thus, all points on the same line through the origin share the same *FFR* and, therefore, cannot be distinguished by the *FFR* alone. So, the *FFR* summarizes the information carried by *P*_*D*_ and *P*_*A*_ “one-dimensionally” along the lines passing the origin in the *P*_*A*_-*P*_*D*_-plot.

This mathematical result provides guidance to answer the remaining question whether the *FFR* can be interpreted as a sound measure of stenosis. As a counterexample, consider a patient developing a stenosis resulting in a decreased post-stenotic pressure, from *P*_*D*_ to γ*P*_*D*_ (direct result of increased stenotic fluid resistance), and a decreased aortic pressure, say from *P*_*A*_ to β*P*_*A*_ (indirect result of reduced cardiac performance due to a decreased perfusion of the cardiac muscle tissue). Then the *FFR* changes from *P*_*D*_/*P*_*A*_ to γ*P*_*D*_/β*P*_*A*_, that is a change from *FFR* to (γ/β) *FFR*. Depending on the actual numerical values of γ and β, the *FFR* will decrease (γ < β), remain unchanged (γ = β), or will increase (γ > β) (see Figure 2B). Clearly, this ambiguity of the *FFR* is an undesired and unanticipated property for a sound measure of degree of stenosis.

The common clinical experience of a decreasing *FFR* with a worsening of the stenosis, may be explained by the assumption that the decrease in *P*_*D*_ is often larger than the change in *P*_*A*_ (γ < β) and, hence, the *FFR* will decrease with a worsening of the stenosis.

### Relationship Between FFR and Degree of Stenosis

In order to find the relationship between the *FFR* and the degree of narrowing in the stenosed artery, further modeling of the stenosis is required. Various approaches may be chosen: (1) a uniform narrowing of the vessel's cross-sectional area from the normal value *A*_0_ to the “narrowed value” *A*_S_ over the full vessel's length *L* and, then, using Poiseuille's law to calculate the narrowed vessel's hemodynamic resistance; or (2) a local narrowing of the vessel's cross-sectional area from the normal value *A*_0_ to the “narrowed value” *A*_*S*_ over the stenosis length *L*_*S*_ and, then, using Bernoulli's law to calculate the hemodynamic resistance. The first approach will be used in this study.

#### Degree of Stenosis

Let *A*_*S*_ (*d*_*S*_) and *A*_0_ (*d*_0_) be the cross-sectional area (diameter) of the coronary artery with and without a stenosis. Then, the degree of cross sectional area narrowing (α) is defined as

(8) $$\begin{array}{l} \alpha =\frac{A_{S}}{A_{0}}\text{=}{\left(\frac{d_{S}}{d_{0}}\right)}^{2}\text{, with: 0}\leq A_{S}\leq A_{0}\text{ and }0\leq d_{S}\leq d_{0} \end{array}$$

with α in the range 0 (complete stenosis) to 1 (no stenosis). Alternatively, the stenosis degree *S* is defined as

(9) $$\begin{array}{l} S=1-\frac{d_{s}}{d_{0}} \end{array}$$

Clearly, both measures are related,

(10) $$\begin{array}{l} \alpha ={\left(1-S\right)}^{2}\text{ and }S=1-\sqrt{\alpha } \end{array}$$

The advantage of using α is, however, that the subsequent formulae will be simpler.

#### Poiseuille's Law Applied to a Uniform Narrowed Vessel

Let *R*_*S*_(α) be the hemodynamic resistance (mmHg.s/ml) of the stenotic artery with a narrowing of degree α over the vessel's length *L* (cm). Then, by applying Poiseuille's law, the resistance *R*_*S*_(α) is,

(11) $$\begin{array}{l} R_{S}\left(\alpha \right)=\frac{\Delta P}{F}=\frac{8\pi \eta L}{A_{S}^{2}}=\frac{8\pi \eta L}{A_{0}^{2}}\frac{1}{\alpha^{2}}=\frac{R_{0}}{\alpha^{2}},\\ \text{ with: }R_{0}=\frac{8\pi \eta L}{A_{0}^{2}} \end{array}$$

where Δ*P* is the vessel's pressure difference (mmHg), *F* is the flow (ml/s), η is the viscosity (mmHg.s/cm^2^), *L* is the vessel length (cm), and *A*_*S*_ the cross-sectional area (cm^2^) of the stenotic artery.

#### Pressures and Flow

With reference to Figure 1, the flow (*F*) equals the perfusion pressure *P*_*A*_ – *P*_*V*_ divided by the sum of the two resistances *R*_*S*_*(*α*)* and *R*_*C*_, i.e.,

(12) $$\begin{array}{l} F=\frac{P_{A}-P_{V}}{R_{C}+R_{S}\left(\alpha \right)} \end{array}$$

Moreover, the distal pressure *P*_*D*_ equals the aortic pressure *P*_*A*_ minus the pressure drop over *R*_*S*_(α), i.e., *R*_*S*_(α) *F*. Hence,

(13) $$\begin{array}{l} P_{D}=P_{A}-R_{S}\left(\alpha \right)F \end{array}$$

and by substitution of Equation (12) in Equation (13) yields,

(14) $$\begin{array}{l} P_{D}=P_{A}-\frac{R_{S}\left(\alpha \right)}{R_{C}+R_{S}\left(\alpha \right)}\left(P_{A}-P_{V}\right)\\ \;=\left(1-\frac{R_{S}\left(\alpha \right)}{R_{C}+R_{S}\left(\alpha \right)}\right)P_{A}+\frac{R_{S}\left(\alpha \right)}{R_{C}+R_{S}\left(\alpha \right)}P_{V}\\ \;=\frac{R_{C}}{R_{C}+R_{S}\left(\alpha \right)}P_{A}+\frac{R_{S}\left(\alpha \right)}{R_{C}+R_{S}\left(\alpha \right)}P_{V} \end{array}$$

The dependence of *FFR* upon the degree of narrowing α is found by first the substitution of Equation (14) in Equation (1), i.e.,

(15) $$\begin{array}{l} FFR=\frac{P_{D}}{P_{A}}=\frac{R_{C}}{R_{C}+R_{S}\left(\alpha \right)}+\frac{R_{S}\left(\alpha \right)}{R_{C}+R_{S}\left(\alpha \right)}\frac{P_{V}}{P_{A}} \end{array}$$

and, subsequently, the substitution of Equation (11) in Equation (15), i.e.,

(16) $$\begin{array}{l} FFR=\frac{P_{D}}{P_{A}}=\frac{R_{C}}{R_{C}+\frac{R_{0}}{\alpha^{2}}}+\frac{\frac{R_{0}}{\alpha^{2}}}{R_{C}+\frac{R_{0}}{\alpha^{2}}}\frac{P_{V}}{P_{A}} \end{array}$$

or, equivalently,

(17) $$\begin{array}{l} FFR=\frac{P_{D}}{P_{A}}=\frac{1}{1+\frac{R_{0}}{R_{C}}\frac{1}{\alpha^{2}}}+\frac{\frac{R_{0}}{R_{C}}\frac{1}{\alpha^{2}}}{1+\frac{R_{0}}{R_{C}}\frac{1}{\alpha^{2}}}\frac{P_{V}}{P_{A}} \end{array}$$

Note: The *FFR* in Equations (15) or (16) applies to both *FFR*^(*H*)^ or *FFR*^(*B*)^, depending on whether the pressures were measured under hyperemia or baseline conditions.

### Patient Study

This retrospective sub-study evaluates data from 22 patients (age 67 ± 11 years) from Aalst Cardiovascular Center (Belgium), having right coronary artery (RCA) stenosis in proximal (p, *N* = 8), medial (m, *N* = 12), or distal (d, *N* = 4) part of the vessel. *FFR* was derived from the ratio of the average blood pressure distal to the coronary artery stenosis (*P*_*D*_) and the average pressure in the aorta (*P*_*A*_), both obtained during i.c. adenosine infusion or after an i.v. bolus injection. Technical details are described elsewhere (5). All patients gave permission to use their data in anonymized investigations by signing a consent form. This retrospective study was exempt from institutional review by the Clinical Review Board.

## Results

### *In silico* Study

**Figure 3:**
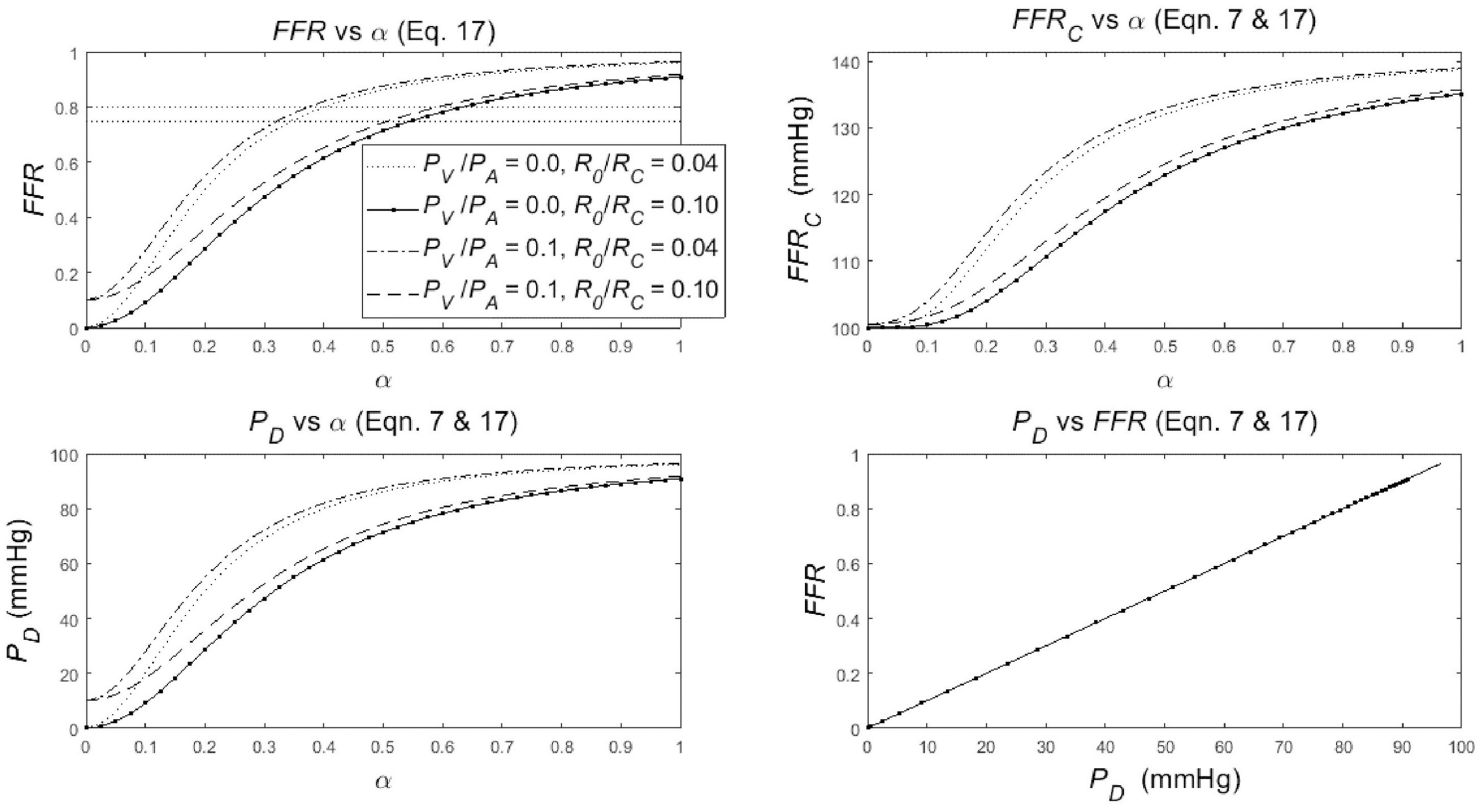
Simulation results for the non-linear dependence of: • the *FFR* with common reference values at 0.75 and 0.8 (upper-left panel), • its companion *FFR*_*C*_ (upper-right panel), • the distal-to-stenosis pressure *P*_*D*_ (lower-left panel), to the degree of stenosis α (α = 1 is no stenosis, α = 0 means complete occlusion) for different values of *R*_0_/*R*_*C*_ and *P*_*V*_/*P*_*A*_ (see legend). • The relation of *FFR* as function of *P*_*D*_ (lower-right panel), (The legend in upper-left panel applies also to the upper-right and lower-left panel). See section Results for details.

The dependence of the *FFR, FFR*_*C*_ and *P*_*D*_ on the degree-of-stenosis α (α = 1 is no stenosis, α = 0 is complete occlusion) is specified in Equation (17) combined with Equation (7) (see section Methods). To discuss the nature of the dependence of *FFR, FFR*_*C*_, and *P*_*D*_ on α, three graphs are created, and an additional graph is drawn to document *FFR* vs. *P*_*D*_ (Figure 3). In the following four points, the merits of each of these graphs is presented in detail:

1. The upper-left panel shows the clearly non-linear dependence of *FFR* upon α (Equation 17), for various settings of the parameters: *R*_0_/*R*_*C*_ = 0.04 or 0.1 with *P*_*V*_/*P*_*A*_ = 0 or 0.1 (see legend in upper-left panel). Note the following:
  - The four lines share a similar S-shape (which is common for a hyperbolic function of the form in Equation 17) but the S-shaped curves start and end at different levels. In particular, the curves start (α = 0) at *P*_*V*_/*P*_*A*_ [i.e., the origin for *P*_*V*_/*P*_*A*_ = 0 and point (0, 0.1) for *P*_*V*_/*P*_*A*_ = 0.1] and the lines end (α = 1) at approximately (1+ *R*_0_/*R*_*C*_)^−1^ (i.e., approximately 0.96 and 0.83, almost independently of *P*_*V*_/*P*_*A*_). Note that *P*_*V*_/*P*_*A*_ dominates the starting values (left) while *R*_0_/*R*_*C*_ dominates the end values (right), resulting in a crossover of the dotted and dashed line. These four example curves can be used to predict other parameter settings. The lower left and upper right point is determined by the value of *P*_*V*_/*P*_*A*_ and (1+ *R*_0_/*R*_*C*_)^−1^ while the steepness of the curve decreases with an increasing *R*_0_/*R*_*C*_. In summary: the dependence of *FFR* on α is an S-shaped relation with the start and end points dependent upon the ratio of the aortic and the venous pressures, as well as the ratio of the non-stenotic arterial and capillary-venous resistances.
  - The S-shaped form of the curves implicates that the change of the *FFR* for a change of α is strongly dependent on the particular location considered. In the steep middle part of the curve, a change of α results in a relatively large change in *FFR*, while a same sized change in α will result in much smaller change in *FFR* in the flat lower and upper parts of the curve. This notion is illustrated by the horizontal line with dots, where the dots are separated by an equal step size in *FFR* while the associated step size in α varies with the steepness of the curve. Thus, the sensitivity of the *FFR*, as a measure of stenotic narrowing, is strongly dependent upon the degree of stenotic narrowing. Technically speaking, this makes that *FFR* is a measure on an ordinal scale (i.e., equal changes in α yield unequal changes in *FFR*). This fact implies that common statistics like means and standard deviations, as well as parametric statistical tests, like Student's *t-*test, are here strictly speaking inappropriate. In summary, a unit change of *FFR* does not correspond to a unit change in α.
  - The venous pressure is of influence on the *FFR*; the larger the *P*_*V*_/*P*_*A*_, the larger the *FFR* although this effect is more pronounced for lower α. This influence may lead to an overestimation of the actual value of the *FFR*. Technically speaking, the *FFR* is a biased measure of α. Similarly, the capillary resistance is of strong influence on the *FFR*; the larger *R*_0_/*R*_*C*_ (i.e., the smaller *R*_*C*_) the less steep the *FFR*-curve is, with as result quite different values of *FFR*. In particular, the *FFR*s as measured during baseline and hyperemia are expected to differ significantly, where the baseline *FFR* is larger than the hyperemic *FFR* [see Equation (6) in section Methods]. In summary, the *FFR* is a biased measure of α, and the uncontrolled bias will present itself as random variations in inter-individual results.
2. The upper-right panel shows a somewhat similar non-linear S-shaped dependence of *FFR*_*C*_ upon α (Equations 7 and 17), for four different setting of the parameters: *R*_0_/*R*_*C*_ = 0.04 or 0.1 with *P*_*V*_/*P*_*A*_ = 0 or 0.1 (see legend in upper-left panel) with *P*_*A*_ = 100 mmHg is all four cases. Comparison of *FFR*_*C*_ with *FFR* shows a marked shape difference for the smaller valued α's, and a completely different *FFR*_*C*_ scale, running from 100 to 140 mmHg, i.e., ranging between *P*_*A*_ and almost *P*_*A*_√2 (see Equation 7). In summary, the dependence of *FFR*_*C*_ upon α is somewhat similar to the dependence of *FFR* upon α.
3. The lower-left panel shows that the dependence of *P*_*D*_ upon α (Equations 7 and 17) is a scaled version of *FFR* for the four different setting of the parameters: *R*_0_/*R*_*C*_ = 0.04 or 0.1 with *P*_*V*_/*P*_*A*_ = 0 or 0.1 (see legend in upper-left panel) with *P*_*A*_ = 100 mmHg is all four cases. The only difference with *FFR* is the fact that the scale runs from 0 to 100 mmHg while the curves start (α = 0) at *P*_*V*_ and end (α = 1) at approximately (1+ *R*_0_/*R*_*C*_)^−1^ *P*_*A*_ (see Equation 14). In summary, the dependence of *P*_*D*_ upon α is a scaled version of the dependence of *FFR* upon α.
4. The lower-right panel shows the dependence of *FFR* upon *P*_*D*_ (Equations 7 and 17), which is exactly a straight line through the origin for the four different settings of the parameters: *R*_0_/*R*_*C*_ = 0.04 or 0.1 with *P*_*V*_/*P*_*A*_ = 0 or 0.1 with *P*_*A*_ = 100 mmHg in all cases. This single straight line is easily explained by rewriting the previous result *P*_*D*_ = *P*_*A*_ *FFR* (Equation 7) as *FFR* = *P*_*A*_^−1^ *P*_*D*_. Now it becomes evident that in a graph this relation is a straight line through the origin with a slope *P*_*A*_^−1^. In summary, the dependence of *FFR* upon *P*_*D*_ is reflected by a straight line.

**Figure 4:**
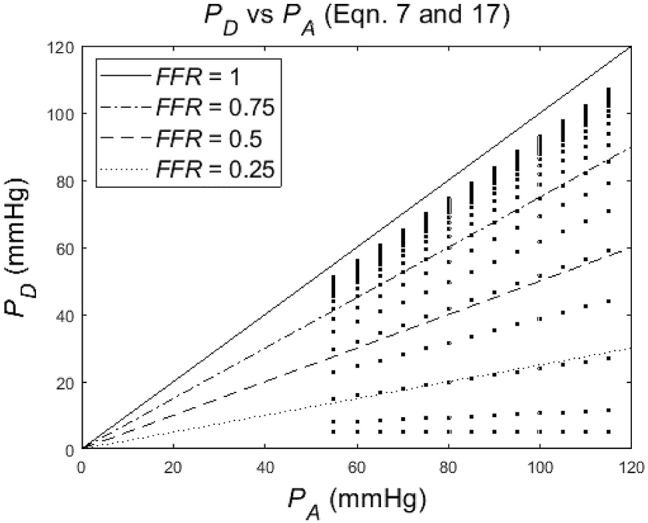
Simulation of distal-to-stenosis pressure *P*_*D*_ vs. mean aortic pressure *P*_*A*_ (Equation 17) with each dot representing a case with *P*_*A*_ running from 55 to 115 mmHg for different values of the degree of stenosis α (α running from 1 to 0), and with lines (Equation 7) indicating *FFR* at 1, 0.75, 0.5, and 0.25.

Figure 4 shows the dependence of *P*_*D*_ on *P*_*A*_ for a particular degree of stenosis α (each dot represents a particular case of values of α, *P*_*D*_ and *P*_*A*_. Note that the distance between the points is increasing or, equivalently, the density is decreasing, for a decreasing α.

### Patient Study

**Figure 5:**
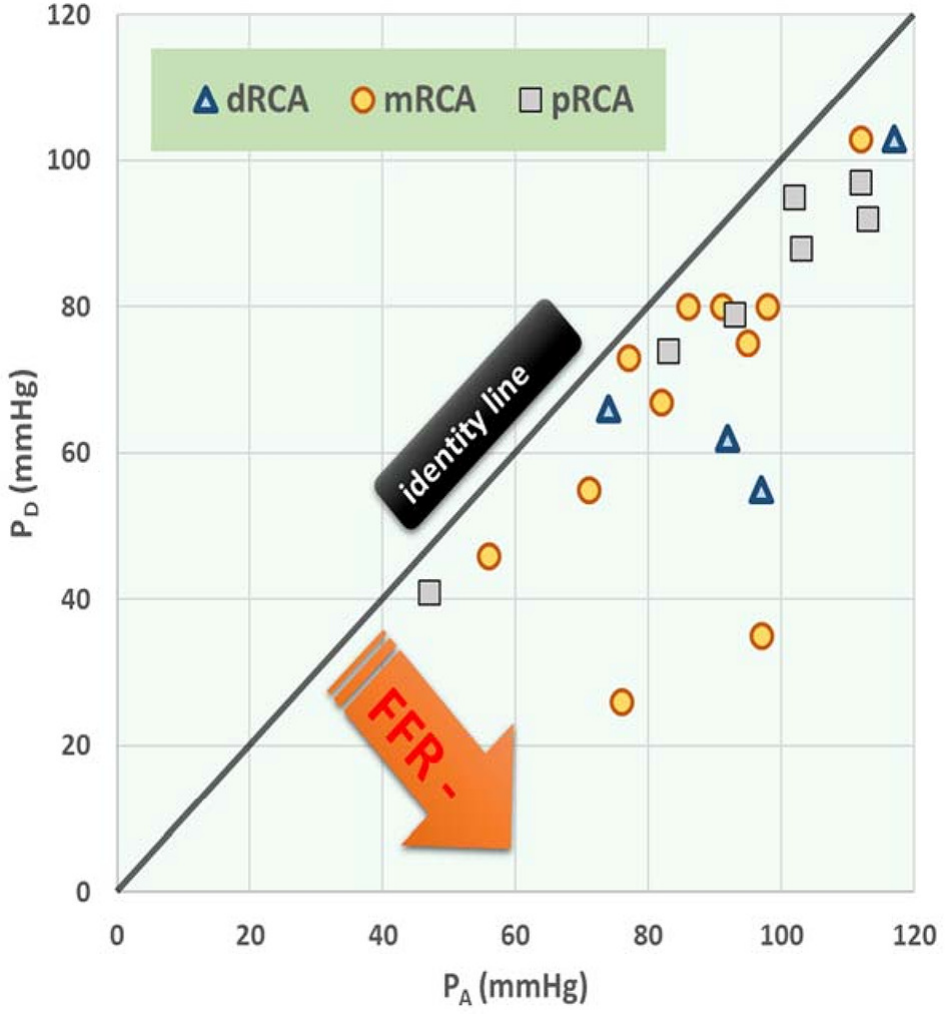
Post-stenotic pressure (*P*_*D*_) vs. mean aortic pressure (*P*_*A*_) during hyperemia with stenosis at different anatomical locations. Data points refer to 22 patients with right coronary artery (RCA) stenosis. The three symbols specify the anatomical location of each stenosis, being distal (d), medial (m), or proximal (p). The black (identity) line corresponds with a fractional flow reserve (*FFR*) of 1.00 (i.e., no stenosis). Regression line: *P*_*D*_ = 0.873 *P*_*A*_ – 6.836, R = 0.728, *P* < 0.001, *N* = 22. In the section Discussion, this graph is compared with Figure 4 which shows results from the *in silico* study.

**Figure 6:**
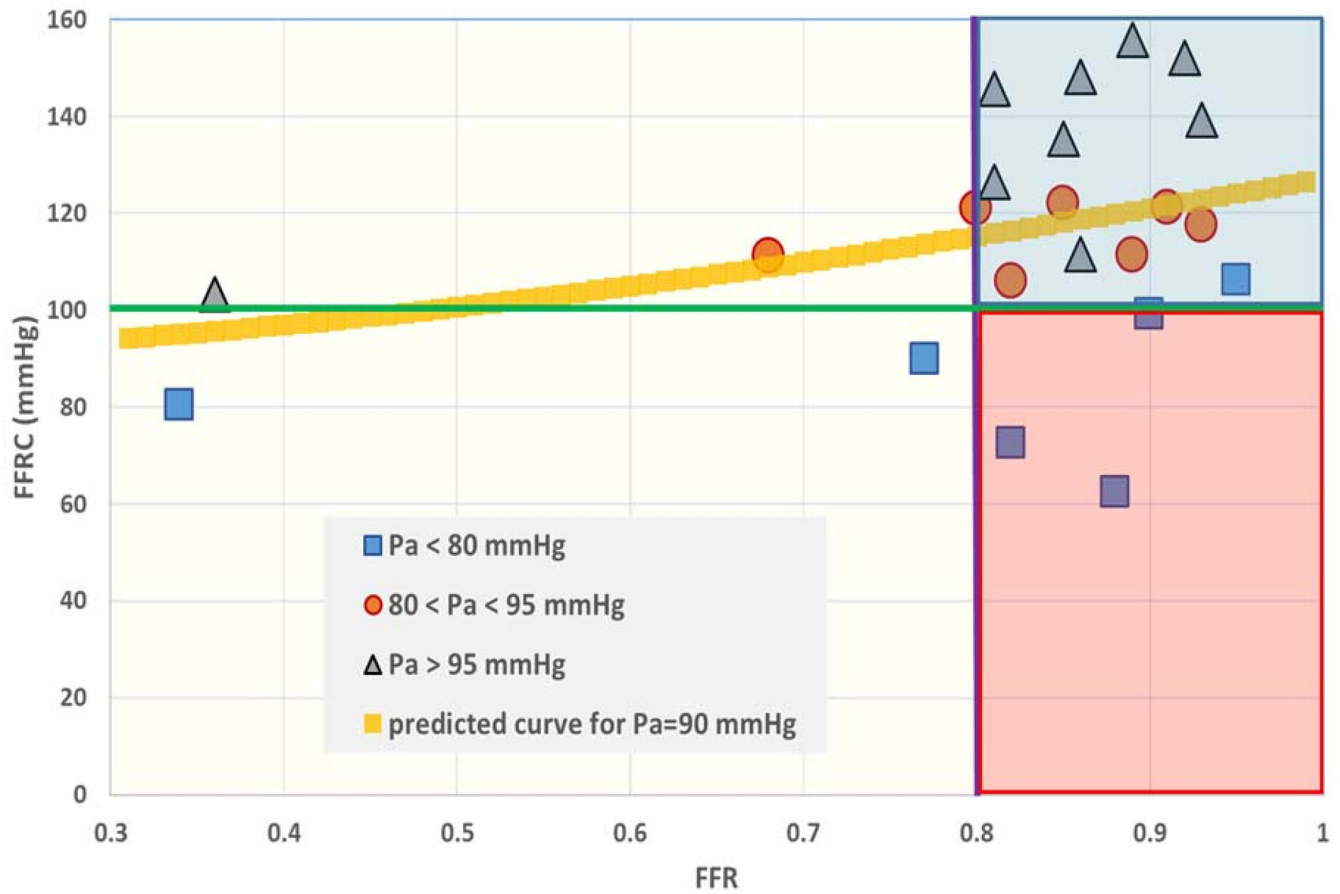
Fractional flow reserve (*FFR*) and the intrinsic companion (*FFR*_*C*_). The interpretation of *FFR* is not unique, as a large spread of the co-metric *FFR*_*C*_ can be discerned. In the past the region between 0.75 and 0.80 was termed the “gray zone.” Data points having FFR > 0.80 (purple line) are often considered to refer to patients who do not require revascularization. However, the situation becomes different if the *FFR* companion (*FFR*_*C*_) is accepted as a second criterion. When, for example, the green line is taken as an additive cut-off level, then the data located in the red shaded area do not simultaneously fulfill both criteria. Obviously, establishment of combined cut-off levels requires robust evaluation in large patient groups. The yellow curve is calculated for *P*_*A*_ = 90 mmHg on the basis of Equation (7), i.e., $FFR_{C}=P_{A}\sqrt{1+FFR^{2}}$.

**Figure 7:**
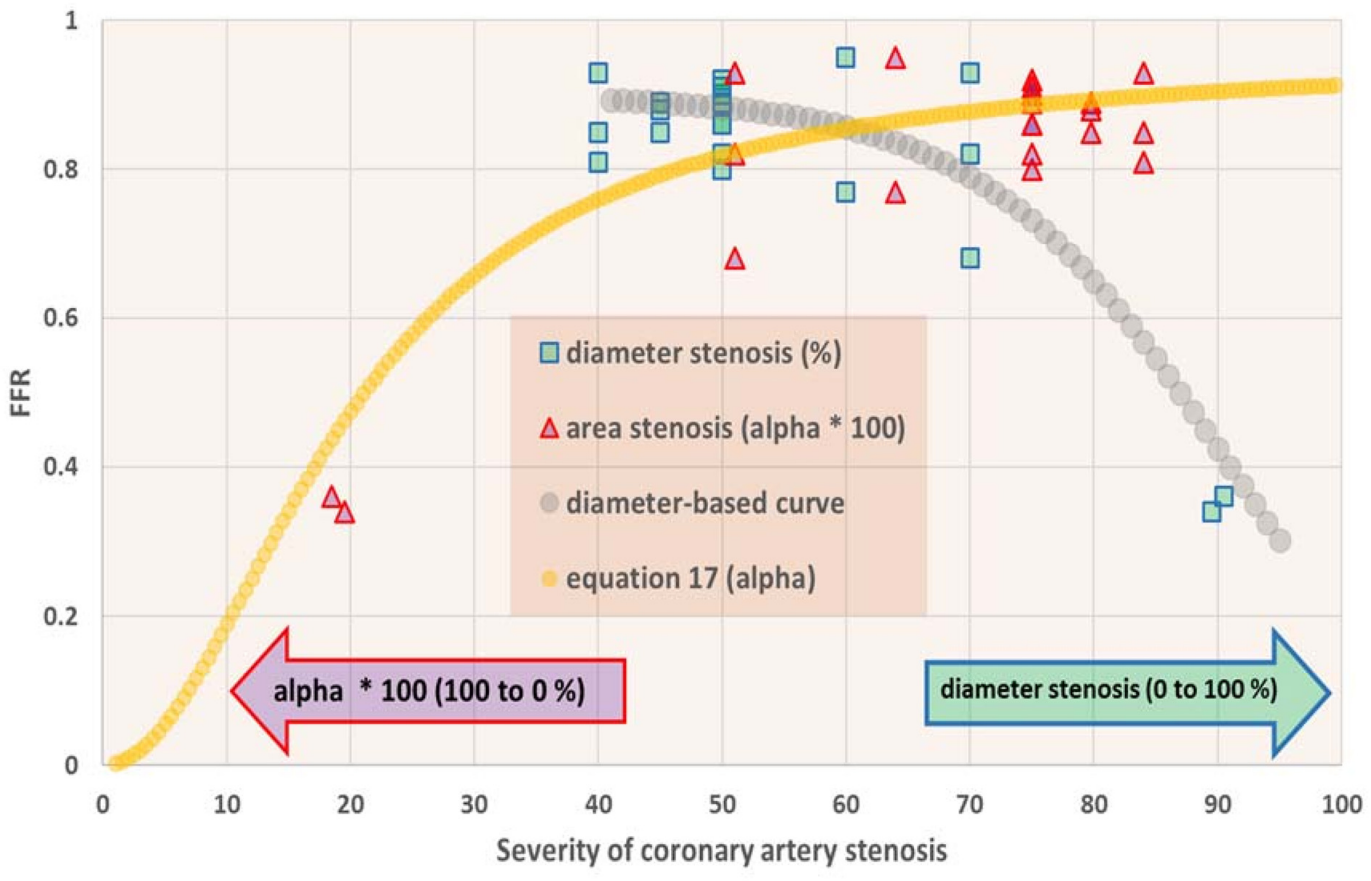
Fractional flow reserve (*FFR*) in dependence of degree of coronary artery stenosis. For the patients studied (*N* = 22), the *FFR* is shown vs. percentage diameter stenosis (squares) as well as vs. the metric α as defined in Equation (8) (triangles). On the abscissa the scales for these metrics run in opposite directions, and therefore all data points are shown twice. Note that *FFR* sharply drops if the stenosis exceeds 70% diameter reduction. The curve for α (Equation 17) shows a sigmoid pattern. In the section Discussion, this graph is compared with results from the *in silico* study (Figure 3, upper-left).

**Figure 8:**
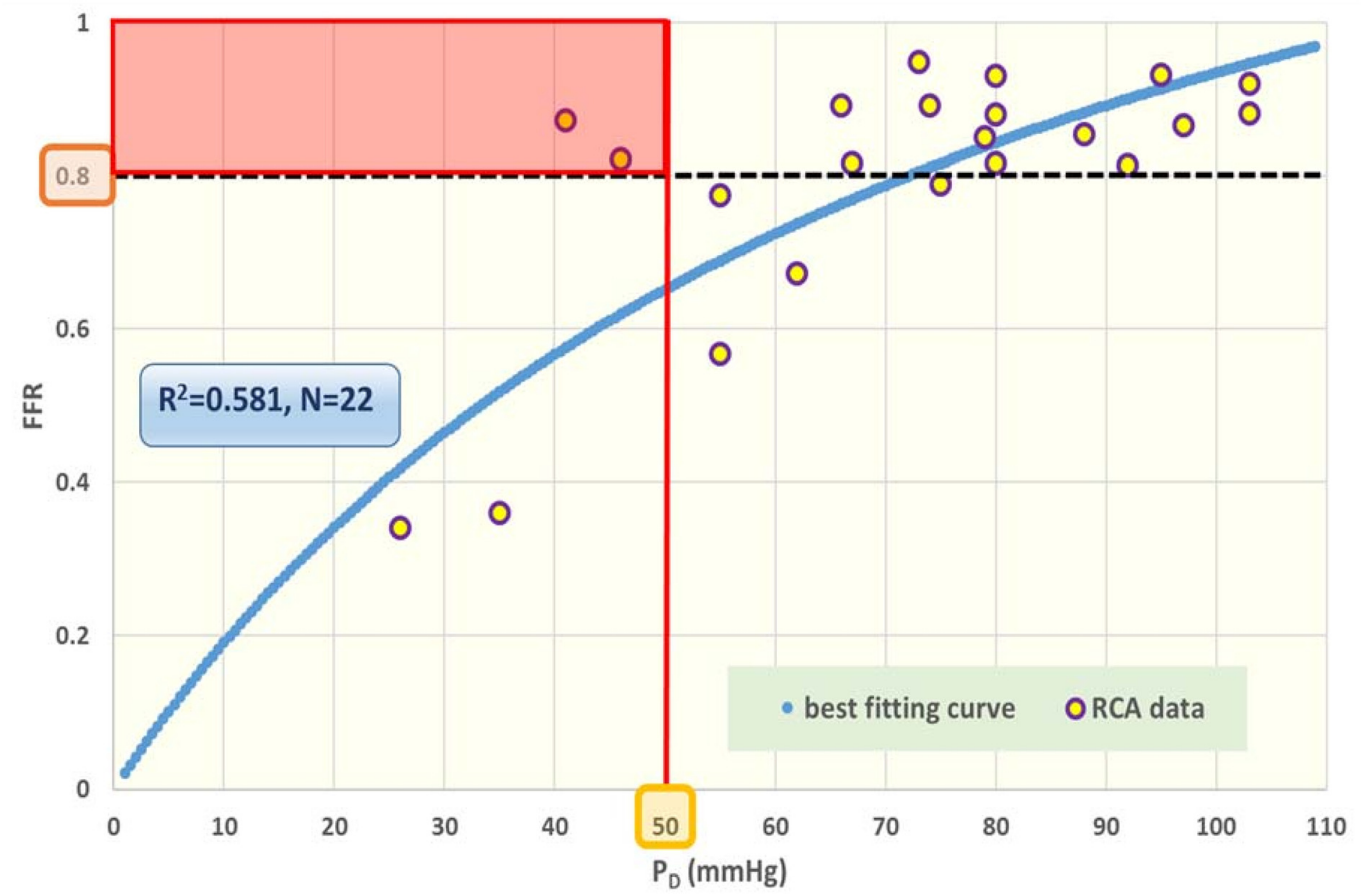
Fractional flow reserve (*FFR*) against post-stenotic mean pressure (*P*_*D*_). Data points for cardiac patients (*N* = 22) with right coronary artery (RCA) stenosis. The blue curve refers to the best fitting regression (*R*^2^ = 0.581), predicted on the basis of the theoretically derived formula *FFR* = *P*_*D*_/(c_1_+c_2_*P*_*D*_). The black broken line indicates the traditional cut-off level at 0.80 for *FFR*. The various data points near this line can be further characterized by specifying the prevailing *P*_*D*_ value. The red line reflects a tentative complementary cut-off for the co-variable *P*_*D*_, assuming that a driving *P*_*D*_ below 50 mmHg is inadequate for appropriate perfusion. Acceptance of this criterion implies that the patients within the red rectangular area are in jeopardy. Clearly, any suggested combination of cut-off levels requires future robust evaluation in large patient groups. In the section Discussion, this graph is compared with results obtained from the *in silico* study (Figure 3, lower-right).

The distribution of *P*_*A*_ and *P*_*D*_ data pairs is presented in Figure 5. The spread of *FFR*_*C*_ for the recorded *FFR* values is shown in Figure 6, while Figure 7 illustrates that *FFR* sharply declines in a non-linear manner when the diameter reduction decreases beyond 60%. Note that in this study 2 out of 3 data pairs indicate that *FFR* can still be above the 0.80 cut-off level, while the associated diameter reduction is as large as 70%. Also is shown that the cross sectional area based stenosis metric α (running in opposite direction along the abscissa) follows a sigmoid pattern, as theoretically predicted (Equation 17). Figure 8 presents *FFR* against mean pressure as measured distal from the coronary stenosis, using adenosine induced hyperemia. The blue curve refers to the best fitting regression (yielding *R*^2^ = 0.581), calculated on the basis of the theoretically derived formula *FFR* = *P*_*D*_/(c_1_+${\text{c}}_{2}^{*}$P_*D*_) described elsewhere (11). This approach ensures that the theoretical point where the value for *FFR* vanishes occurs at *P*_*D*_ = 0 mmHg, while *FFR* also asymptotically approaches the upper limit of 1.0 as *P*_*D*_ increases to its physiological maximum. *FFR*_*C*_ vs *P*_*D*_ yields R = 0.91.

### Evaluation of FFR Data Presented in the Literature

**Figure 9:**
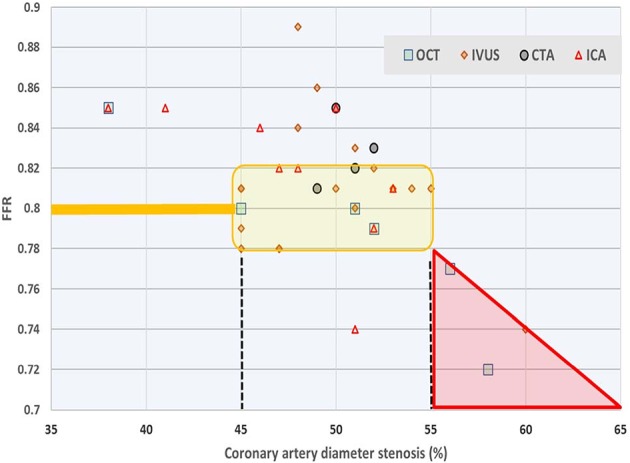
Survey of relationship between FFR and diameter stenosis. Average values reported in 38 studies on the basis of four measurement techniques: optical coherence tomography (OCT), intravascular ultrasound (IVUS), computed tomography angiography (CTA), and intracoronary angiography (ICA). Most authors employ linear regression or fit a second order polynomial for their study participants. The yellow shaded area refers to values of 0.78 ≤ FFR ≤ 0.82 around the common cut-off point, which narrow range corresponds with (averaged) diameter reduction anywhere between 45 and 55%. Data from Chu et al. (17).

As the relationship between FFR and the degree of stenosis is the main subject of this study, we also collected a variety of data from the literature. In a computational fluid dynamics (CFD) modeling study (12) it was shown that uncertainty in minimum lumen diameter had the largest impact on hemodynamic simulations, followed by boundary resistance, viscosity and lesion length. Also, uncertainties were not additive, and only slightly higher than the highest level found for a single parameter. Also based on CFD and using angiographic images it was demonstrated (13) that sensitivity analysis for physiological lesion significance was influenced less by coronary or lesion anatomy (33%) than by microvascular physiology (59%). Using a reduced-order model for the estimation of *FFR* (rather than 3D) based on blood flow simulations that incorporated clinical imaging and patient-specific characteristics, others found that model errors were small, and that uncertainty related to the factor by which peripheral resistance is reduced from baseline to hyperemic conditions proved to be the most influential parameter for *FFR* predictions, whereas uncertainty in stenosis geometry had greater effect in cases with low *FFR* (14). Similarly, 296 lesions were studied (15) and the authors compared (by linear regression) various clinically relevant measures, including diameter stenosis (*R* = 0.565), lesion length (*R* = 0.306), reference vessel cross sectional area (*R* = 0.195), and the myocardial supply area subtended by the coronary vessel under study (*R* = 0.504). In an attempt to further simplify calculations a 1D model was compared with a 3D model, and found to yield nearly similar findings for *FFR* (16). Findings reported in 38 studies (17) are summarized in Figure 9 which further illustrates the discrepancy between *FFR* and diameter-based indicators of coronary luminal obstruction.

### Clinical Implications

For coronary arteries we analyzed the relationship between local diameter stenosis and the associated pressure gradient using a simple mathematical model. In clinical practice the pre- and post-stenotic pressures are obtained during induced hyperemia, and the key metric *FFR*, calculated for medical decision making, considers the minimum value of the ratio of these two pressures. We derived that the resulting dimensionless ratio requires consideration of an associated companion *FFR*_*C*_, which is the Pythagorean mean of the two pressures involved. Similar considerations apply to the separate category of coronary flow reserve metrics, as well as to recently introduced alternative metrics such as adenosine-free *P*_*D*_*/P*_*A*_ and wave-free approaches. As on theoretical grounds any companion (as defined in our study by the pertinent hypotenuse) may not be neglected just for simplicity or convenience reasons, it is necessary to evaluate the precise clinical impact of *FFR*_*C*_ in large scale patient studies.

## Discussion

Limitations of myocardial perfusion, due to coronary arterial stenosis, are best described by pressure-flow relationships. In clinical practice, such investigations are limited to the estimation of either coronary artery diameter, pressure, or flow. Historically diameter reductions were calculated from coronary angiograms with emphasis on anatomy. Subsequent analysis referred to physiology and was based on (surrogates of) flow measurements aimed at determination of the reserve capacity, i.e., the maximum flow increase during hyperemia. One of the most popular approaches does not directly measure flow, but rather the ratio of two pressures measured proximally and distally from the stenosis during hyperemia, and is referred to as *FFR* (the primary measure evaluated in this study). Next simplified versions were explored, including ratios obtained during the (wave-free) diastolic phase (18), and even ratios without induction of hyperemia. Agreements and differences among resting coronary physiological indices led to the query: *Are all things equal?* (19). Recently, prudent thoughts were formulated regarding comparisons of various techniques, while pointing to the question what is precisely compared with what, and that question was formulated against the background and role of the acclaimed “gold standard” (20). Therefore, the aim of our study was a model-based evaluation of the *FFR*, because the model provides a complete knowledge and a full control (“gold standard”) of the conditions. Moreover, the model enables the detailed evaluation of the characteristics of the *FFR*, although *in silico*. Then, the *in silico* study outcomes have been compared with patient data regarding coronary diameter (reduction), pressures proximal and distal to the stenosis during baseline and adenosine. So, the *in silico* study is used to generate predictions that are subsequently verified using available clinical data.

The outline of the discussion is as follows: first, the answers to our four research questions (see section Introduction) are discussed point-by-point; secondly, the *in silico* study outcomes are compared with clinical data; thirdly, the results of our study are put into the perspective of other model studies; finally, the limitations of our study are discussed.

The *FFR* was evaluated as a measure of arterial coronary stenosis by using a simple mathematical model of the coronary system. The coronary circulation was modeled with two Poiseuillian hemodynamic resistances, one for the arterial part and one for the capillary and venous part and an aortic and venous pressure (see Figure 1), all in close correspondence with the original approach (7). The arterial stenosis was described by reduction of cross-sectional surface in Poiseuilles law (Equations 8–11). This simple model allowed the calculation of explicit formulae (with graphs) for the flow *F* (Equation 12), the distal-to-stenosis pressure *P*_*D*_ (Equation 14), the *FFR* (Equation 17), all as a function of the degree of stenosis α. This model and these formulae and associated graphs, allow the evaluation of the *FFR* as a measure of arterial stenosis. First, our main objectives in this study (see section Introduction) are discussed point-by-point:

- Firstly, the *FFR's definition and its theoretical consequences*. The *FFR* is defined as the ratio of the maximum myocardial blood flow in presence of a stenosis and the theoretical maximum myocardial blood flow an absence of the stenosis. Pijls and De Bruyne argued that the intended *FFR* can be approximated by the ratio of the mean distal-to-stenosis pressure and the mean aortic pressure, both measured during a drug-induced hyperemia. Our analysis confirmed, not surprisingly, the Pijls and De Bruyne results but also clearly showed that: (1) the *FFR* is not a simple linear measure of the degree of stenosis (Figure 3 upper-left panel); and (2) the FFR measured at baseline conditions and during hyperemia are related similarly to the degree of stenosis and, as expected, the *FFR* is larger at baseline than in hyperemia *FFR* (see Equation 6).
- Secondly, the *relation between the FFR and the degree of coronary arterial stenosis* was identified. This relation was found to be an S-shaped curve, possibly significantly influenced by both the size of the capillary resistance relative to the stenotic resistance and the size of the venous pressure relative to the aortic pressure. The S-shaped curve implicates that the sensitivity of the *FFR*, as a measure of stenotic narrowing, is strongly dependent upon the degree of stenotic narrowing itself. Technically speaking, this makes *FFR* a measure of α on an ordinal scale (i.e., equal changes in α yield same-direction but unequal changes in *FFR*) implying that common statistics like means and standard deviations and, parametric statistical tests, like Student's *t-*test, are strictly speaking inappropriate. The influence of the venous pressure may lead to an overestimation of the actual value of the *FFR*. Technically speaking, the *FFR* is a biased measure of α. The influence of the capillary resistance on the steepness the *FFR*-curve changes the sensitivity of the *FFR*, resulting in quite different values of *FFR*. In particular, the *FFR* values measured during baseline and hyperemia are expected to differ significantly, with the baseline *FFR* larger than the hyperemic *FFR* (see Equation 6). All these influences make the *FFR* a biased measure of α and these uncontrolled biases will present themselves as random variations in intra- and inter-individual clinical results.
- In addition, one needs to consider the following trade-off in answering the question whether to determine the *FFR* under hyperemic or baseline conditions: In the upper-left panel of Figure 3, the two upper and two lower curves can be interpreted as the *FFR* at baseline and at hyperemic conditions, respectively (using the following rationale: the *R*_0_/*R*_*C*_ value for the lower lines are larger than for the upper lines. Thus, by the inverse proportionality of *R*_*C*_, the *R*_*C*_ values of the upper lines are larger than for the lower lines, and therefore the upper and lower lines refer to the baseline and hyperemic conditions, respectively). The disadvantage of the “baseline lines” (i.e., the upper lines) over the “hyperemic lines” (i.e., the lower lines) is that these “baseline lines” are far more curved than the “hyperemic lines.” So, the sensitivity of the *FFR* for changes for α is expected to be more uniform in the “hyperemic lines.” However, the disadvantage of the “hyperemic lines” (lower lines) is that the biasing influence of the venous pressure (*P*_*V*_) is more pronounced compared to the “baseline lines” (upper lines); notice that the distance between the lower “hyperemic lines” is larger compared to the distance between the higher “baseline lines.” The relevance of these findings—a more curved hyperemic line vs. a more pronounced influence of venous pressure—is in need of a clinical evaluation study, in particular for the region around the reference value *FFR* = 0.80. In addition, the difference between the baseline and hyperemic lines in the graph indicates that the reference value for *FFR* needs to be chosen significantly different for the hyperemic and baseline conditions.
- Thirdly, the *FFR* is *a summary* of two pressures, *P*_*D*_ and *P*_*A*_, in one ratio, *P*_*D*_/*P*_*A*_. Our analysis (Figure 2) showed an ambiguous interpretation of the *FFR*. That is, a decrease (increase) of the *FFR* not necessarily results from an increase (decrease) of the degree of stenosis. In fact, an unambiguous interpretation of the *FFR* is only possible under the extra condition of a constant arterial pressure *P*_*A*_. This is a somewhat surprising finding because intuitively one expects the *FFR* to be controlled for variation in *P*_*A*_ by the fact that the *FFR* normalizes *P*_*D*_ to *P*_*A*_. In conclusion, in the present clinical experience with *FFR* the decrease in *P*_*D*_ may be larger than in *P*_*A*_ and, hence, the *FFR* will decrease with a worsening of the stenosis and the disturbing and ambiguous influence of varying *P*_*A*_ is interpreted as random variations (noise). As a suggestion for further clinical research, the relative contribution of *P*_*D*_ and *P*_*A*_ on *FFR* can be easily assessed in clinical data by taking the logarithm of the *FFR*, i.e., ln(*FFR*) = ln(*P*_*D*_) – ln(*P*_*A*_) and, then performing a linear regression analysis to the line ln(*FFR*) = A^*^ln(*P*_*D*_) – B^*^ln(*P*_*A*_) + *C*; the size and significance of parameters *A* and *B* indicate the relative importance of *P*_*D*_ and *P*_*A*_ to *FFR*.
- Fourthly, given the complex dependence of the *FFR* on the degree of stenosis and the additional biasing influences of the venous pressure and capillary resistance, one might wonder whether the *FFR* as pressure ratio can be improved. *To hint for an alternative*: clearly the stenotic pressure drop, i.e., *P*_*A*_ – *P*_*D*_ in the model (Figure 1) is of key importance, but needs to be compared with the pressure drop over the capillary and venous part of the circulation, i.e., *P*_*D*_ – *P*_*V*_. Hence, an obvious choice seems to define the alternative *FFR* as (*P*_*A*_ – *P*_*D*_)/(*P*_*D*_ – *P*_*V*_), which equals (*F R*_0_(α))/(*F R*_*C*_) = *R*_0_/*R*_*C*_ 1/α^2^ (by Equation 11) or, reversely, by rewriting to get α at the left side, the measure of the degree of stenosis is α = √{*R*_0_/*R*_*C*_ (*P*_*D*_ – *P*_*V*_)/(*P*_*A*_ – *P*_*D*_)}. Although this alternative provides explicitly the degree of stenosis and is free of a biasing influence of the venous pressure, this alternative still suffers from the influence of the intra- and inter-individually varying baseline stenotic and capillary resistances. Probably this is a drawback of all attempts to characterize stenotic resistances by a measure based on pressure measurements alone. Fundamentally, limitations of myocardial perfusion due to arterial coronary stenosis are best described by pressure-flow relationships but, in clinical practice, such investigations are often limited to the estimation of coronary artery diameter, pressure, or flow. So, the best practice needs to be found by a mathematical-physical approach, further guided by a subsequent clinical evaluation of stenotic measures.

This completes the discussion of our main objectives regarding the *in silico* study.

The comparison of the outcomes of the *in silico* study with the clinical patient data regarding coronary diameter (reduction) yields the following results:

- The *in silico* model predicts a relation of *FFR*_*C*_ = $P_{A}\sqrt{\left(1+FFR^{2}\right)}$ (see Equation 7). Indeed, the clinical data in Figure 6 reveals such a quadratic relation, but with a large amount of scatter due to inter-individual variation of *P*_*A*_.
- The *in silico* model predicts a S-shaped dependence of *FFR* on α (Figure 3, upper-left panel). Indeed, the clinical data in Figure 7 shows the upper part of the S-shaped form, while the lower part of the S-shape (severe stenosis) is not visible in Figure 7 simply because these severe cases of stenosis are not present in our clinical data set. So, the clinical data is in accordance with the *in silico* model prediction. Note that different measures of stenosis are used. Figure 7 shows both the stenotic diameter reduction and α, while in Figure 3 the free lumen area based metric α is used.
- The *in silico* model predicts, for an increasing degree of stenosis, a decreasing density of cases in the plot of *P*_*D*_ vs. *P*_*A*_ (see Figure 4) and, indeed, this is observed in the clinical data of Figure 5.
- The *in silico* model predicts a linear relation between *FFR* and *P*_*D*_ with a slope *P*_*A*_^−1^, for the case of a constant *P*_*A*_ (that is a straight line from the lower-left corner to the upper-right corner). In Figure 8 the clinical data indeed shows this relation in presence of a large amount of scatter due to inter-individual variation of *P*_*A*_. Based on the calculation of c_1_ and c_2_ (derived from Figure 5) a best fitting curve (blue) was constructed. If a tentative second criterion (*P*_*D*_ cut-off e.g., at 50 mmHg) is applied, then the data points in the red shaded rectangular are do not meet both requirements. This choice implies that two patients are judged to have a functional coronary stenosis despite the fact that their *FFR* > 0.80. Obviously, this approach assumes that the cut-offs for *FFR* and *P*_*D*_ are independent. Therefore, it is very well-conceivable that the criterion for *FFR* may vary with the prevailing *P*_*D*_ level. Based on machine learning methods we have already demonstrated the applicability of a non-linear divider when analyzing ejection fraction in heart failure patients (21).
- For *FFR*, a “gray zone” has been discussed in the literature with values between 0.80 and 0.75 (1). Although this range covers only 5% of the complete theoretical range, the more important issue is the fact that a substantial portion of patients is located within this range of uncertainty. This completes the confirmation of the *in silico* study predictions by our clinical data.

To put our study in perspective: Various modeling approaches have been employed to evaluate the severity of coronary stenosis (22). Some investigators (23) applied numerical modeling of the flow in a stenosed coronary artery in relation to main hemodynamic parameters. Using a resistive model of an epicardial stenosis (0–80% diameter reduction) in series with the coronary microcirculation at maximal vasodilation, *FFR* was evaluated for changes in coronary microvascular resistance (0.1–0.6 mmHg.min/ml), aortic pressure (between 70 and 130 mmHg), and coronary outflow pressure (0–15 mmHg), and it was found that the sensitivity of *FFR* to these hemodynamic changes was highest for stenoses of intermediate severity (23). Recent studies employ either a patient-specific lumped-parameter model of the coronary circulation (9) or applied the SimVascular Cardiovascular Modeling Package (24). Meta-analysis of *FFR* vs. quantitative coronary angiography and non-invasive imaging for evaluations of myocardial ischemia resulted in relatively poor concordance among outcomes (22). Furthermore, a visual-functional mismatch has been reported between coronary angiography and *FFR* (25). Pellicano et al. documented that angiography derived expressions for *FFR* matched those using traditional pressure ratios, thus claiming to integrate anatomy and physiology (26).

In contrast, our investigation concerns *in silico* studies, combined with actual patient data for the RCA; the characteristics (i.e., the scale property and the bias) of the *FFR* are described as a man-made measure (technical term estimator) of arterial coronary stenosis in a simple resistive model of the coronary circulation similar to the original model used by Pijls and De Bruyne. The higher *FFR* sensitivity for stenoses of intermediate severity was confirmed (23). Moreover, the profound influence of venous pressure was emphasized but the main difference with earlier approaches is the introduction of *FFR*_*C*_ as a co-measure of *FFR*. Our graphical-mathematical analysis (with use of Cartesian and polar coordinates) indicates clearly that summarizing two pressures (*P*_*D*_ and *P*_*A*_) in one ratio (*FFR*) only partly captures the information actually collected, and that the complementary information contained in the companion *FFR*_*C*_ appears to be clinically relevant. As a provocative example: Consider the case of an *FFR* = 0.80 calculated form *P*_*D*_ = 40 mmHg, and a worrisome low *P*_*A*_ = 50 mmHg. This situation implies that the patient is both hypotensive (27) and that the perfusion pressure is low. Yet, the *FFR* is not abnormal. One would object, of course, that the *P*_*D*_ and *P*_*A*_ pressures themselves are clear warning signs. But that is precisely the point we emphasize, as their ratio (the *FFR*) is an inadequate summary of two separately already relevant pressures. One must take into account both pressures, or the combination of *FFR* and the *FFR*_*C*_ to acquire the full picture. Only under the very restrictive condition that the *FFR*_*C*_ is constant, the *FFR* is an unambiguous measure of the degree of stenosis. In summary, our investigation evaluates the characteristics of the *FFR* as measure of the degree of stenosis; our main conclusion is that the *FFR* is insufficient a measure of stenosis because: (1) the *FFR* (without *FFR*_C_) cannot be interpreted unambiguously; (2) the *FFR* is on ordinal scale (unit differences in *FFR* are not proportional to unit changes in stenosis with as result that standard statistics, like means, standard deviations, Student's *t*-test) do not apply and non-parametric methods must be applied; (3) the uncontrolled influences of venous and aortic pressure and the capillary resistance on the *FFR* present themselves in the final results as random variations (noise) while, factually, these variations originate from imperfections of the *FFR* as metric.

Pressure loss across a stenosis is a function of resistance, whose components include morphologic factors (including stenosis entrance angle, orifice configuration, length of stenosis, exit angles) as well as physiologic factors such as flow and associated myocardial supply area (28). Recently, the incremental value of also considering the subtended myocardial mass for identifying *FFR*-verified ischemia was confirmed using quantitative CT angiography (29). Furthermore, as explicitly formulated in an editorial, the question arises “*which of the two instruments for gauging stenosis, FFR or angiography, is at fault*” (30). Given the rather constant diameter (among comparable individuals) of the unaffected vessel (which variable is the rather constant number in the denominator for % diameter reduction), it would seem that changes for the pertinent hypotenuse, here associated with diameter reductions due to occlusion, are less pronounced compared to the hypotenuse variation associated with *FFR* determinations, as *P*_*A*_ (which is the denominator in *FFR*) is subjected to a wide range of variations.

This completes the discussion of our analysis against the background of studies which employ *FFR* as a gold standard to evaluate functional limitations associated with epicardial coronary artery stenosis.

## Limitations

Our model-based evaluation of the *FFR* as measure of the degree of stenosis was based on the simple model of the coronary circulation originally used (7). As a result, our study is limited because of (1) the use of Poiseullian resistances in a model that neglects the influences of a non-Poiseuillian pressure-flow relation in the coronary arteries, (2) the neglect of neural and hormonal factors and the autoregulation in the microvascular bed (prearterioles), (3) the neglect of the geometry of the coronary tree, (4) sex-specific differences, extensively reported in the literature (2, 31), were not investigated. Preliminary analysis showed that our approach is still feasible to arrive at similar results for the more complicated cases with non-Poiseuillian and autoregulatory effects on resistance. Moreover, the present model's focus is on hemodynamic resistances only while neglecting the Windkessel dynamics of the coronary system, but a preliminary analysis shows that similar results are found by using a model including the Windkessel properties, yet the manuscript's margin is too small to provide details.

Also, it must be noted that all studies relating *FFR* to relative stenosis severity, including our own investigation, compare two dimensionless ratio-based metrics (32). Such comparisons neglect the corresponding companion metrics. While both constituents of *FFR*_*C*_ may assume a wide range of values (see e.g., Figure 5), it can be stated that the denominator term in the α or S% metrics have a rather fixed value for each vessel, given any particular patient while taking into account body mass and sex (31). The rather fixed reference level in case of diameters or areas clearly renders a more insightful interpretation to this sub-group of ratios.

The *FFR* approach is limited from a technical point of view, as it only considers hyperemic data. Inclusion of baseline values recorded for *P*_*A*_ and *P*_*D*_ may assist in developing a more comprehensive characterization of myocardial perfusion abnormalities.

## Conclusions

The dependence of the *FFR* on the degree of stenosis shows an S-shaped form. Consequently, *FFR* is a measure of the ordinal scale. Moreover, the marked disturbing influences of the aortic and venous pressures and the capillary resistance on the *FFR* will be significantly manifested as random variations (noise) in intra- and inter-individual clinical results. These problems are partly caused by the neglect of the *FFR*'s companion, namely the *FFR*_*C*_ (32). Taken together, the combined use of *FFR* and *FFR*_*C*_, or alternatively *P*_*D*_ and *P*_*A*_ when considered in unison, provide more complete information on a flow limiting coronary stenosis. When analyzing ratios, it may also be useful to consider a logarithmic transformation.

## Ethics Statement

This retrospective study in patients from Cardiovascular Center, Aalst, was exempt from permission, as stated by the local ethics committee. All patients provided permission to use their data for investigational purposes.

## Author Contributions

TF designed the study, developed the software for the simulation studies, and wrote the initial version. RM developed the first versions of the software for the simulation studies and contributed to the text. GH collected patient data and implemented clinical background. PK designed the study, illustrated clinically relevant aspects, and complemented the text.

### Conflict of Interest

The authors declare that the research was conducted in the absence of any commercial or financial relationships that could be construed as a potential conflict of interest.

## Abbreviations

α: degree of cross sectional area narrowing for a coronary artery stenosis
A: cross-sectional area
B: baseline
C: vascular compliance
CFD: computational fluid dynamics
*F*: myocardial blood flow
*F*_*N*_: non-stenotic flow
*FFR(*_*C*_*)*: fractional flow reserve (companion)
H: hyperemia
*L*: full length of a coronary vessel
*L*_*S*_: length of local stenosis
L: blood mass inertance
N: non-stenotic case
cf.: S
*P*_*A*_: coronary pressure proximal from the stenosis
*P*_*D*_: coronary pressure distal of the stenosis
*P*_*V*_: venous pressure
R: Poiseuille resistance
*R*_*A*_: hemodynamic resistance (mmHg.s/ml) of the arterial part
*R*_*C*_: hemodynamic resistance (mmHg.s/ml) of the capillary and venous parts
*R*_0_: hemodynamic resistance (mmHg.s/ml) of the coronary artery without stenosis
RCA: right coronary artery
S: stenosis
cf.: N
S (%): diameter-based percentage of stenosis
cf.: α.
